# Exploring the adoption of diaphragm and lung ultrasound (DLUS) by physiotherapists, physical therapists, and respiratory therapists: an updated scoping review

**DOI:** 10.1186/s13089-025-00412-w

**Published:** 2025-01-20

**Authors:** Simon Hayward, Camella Cardinael, Chloe Tait, Michael Reid, Andrew McCarthy

**Affiliations:** 1 Physiotherapy Department, Blackpool Teaching Hospitals NHS Foundation Trust, Whinney Heys Road, Blackpool, FY3 8PY UK; 2 Acute Medicine Physiotherapy, Lancashire Teaching Hospitals NHS Foundation Trust, Sharoe Green Lane, Fulwood, Preston, PR2 9HT UK; 3 Library and Knowledge Services, Blackpool Teaching Hospitals NHS Foundation Trust, Whinney Heys Road, Blackpool, FY3 8PY UK

**Keywords:** Diaphragm, Lung, Ultrasonography, Physiotherapist, Physical therapist, Respiratory therapist

## Abstract

**Background:**

The adoption of diaphragm and lung ultrasound (DLUS) by physiotherapists, physical therapists, and respiratory therapists (“therapists”) to examine and assess the diaphragm and lungs continues to grow. The aim of this updated scoping review is to re-explore and re-collate the evidence around the adoption of DLUS by therapists.

**Methods:**

This scoping review followed the PRISMA-ScR guidelines. Data sources searched included AMED, EmCare, CINAHL, Embase, Medline, PubMed and Pedro. Grey literature sources were searched alongside communication with leading authors in the field. The Participants, Concept and Context (PCC) approach was employed to formulate the research question. A charting form was developed and piloted to extract: title, authors, year of publication, country of origin, professional group involved (population), lung or diaphragm ultrasound (concept), evaluation method, educational, clinical or research setting (context), subject/disease/patient group, sample size, study design and professional group performing DLUS.

**Results:**

133 studies met all inclusion criteria, an increase of 107 new studies compared to the original scoping review searches 7-years ago. Studies were included from 17 new countries and included 17 new participant populations. Lung ultrasound saw the largest increase in study number with education and implementation emerging as a new area of investigation. Full list of included studies is provided in Supplementary File 1.

**Conclusion:**

The number of DLUS studies involving therapists continues to show international growth with studies investigating an increasing range of participant populations. Published studies now include research on DLUS adoption, implementation, and utility amongst all three of the therapy professions who use DLUS. The potential of DLUS and its direct impact on patient outcomes still needs to be explored further. However, DLUS remains a novel and innovative imaging technique in the hands of physiotherapists, physical therapists, and respiratory therapists as its utility continues to grow in various research, clinical and educational settings.

**Supplementary Information:**

The online version contains supplementary material available at 10.1186/s13089-025-00412-w.

## Background

There is an increasing body of evidence reporting on the adoption, implementation and utility of diaphragm and lung ultrasound (DLUS) within the international physiotherapy, physical therapy, and respiratory therapy communities (for the purposes of this review collectively termed “therapist”) [1]. Therapists from Australia [2], China [3], the Kingdom of Saudi Arabia [4] and the United Kingdom [5] have explored the potential applications, training options and barriers to DLUS adoption within their respective professions. The growing interest in therapist-performed DLUS is multifactorial and includes the COVID-19 pandemic [6], its enhanced accuracy in the diagnosis of pulmonary conditions such as pneumonia [7, 8], pleural effusions [9, 10], as well as dysfunction of the diaphragm [11–14] and its role in the prognosis of response to non-invasive ventilation treatments [15, 16]. In addition to the accurate identification of respiratory pathologies, therapists can use DLUS to monitor the effectiveness of therapeutic or rehabilitative interventions, guide interventional procedures such as titration of mechanical ventilation and weaning [15–18], and as a research outcome measure [19]. DLUS has the potential to provide therapists with a portable, non-invasive and non-ionising imaging modality to enhance many aspects of their professional practice.

In 2016 the lead author (SH) conducted database searches for the original version of this scoping review published in 2018 [20]. Studies included in this earlier scoping review showed ultrasound professions, such as sonographers or radiologists, performed the DLUS on the therapist’s behalf in most cases. However, this became less common as therapists learnt the DLUS imaging techniques themselves and applied it in their research. The methodology and searches for this scoping review have been updated, improved, and re-run seven years after the original searches were performed. Since the 2016 search results, additional population specific review papers have been published which will aid readers to understand the potential uses of DLUS by therapists for diaphragm [21, 22], lung [23–26], or both [27, 28]. Scoping reviews already exist for the wider adoption of point of care ultrasound for both the physiotherapy [29] and respiratory therapy [30] professions. However, nothing has been published on the wider utility of lung or diaphragm ultrasound since 2018 [20].

The purpose of the review is to update any new evidence in reference to the adoption of DLUS by therapists. This in turn will help to map out how the international therapist community is using DLUS to inform their pathology identification, interventional, therapeutic, rehabilitative and research practice. New sections aim to explore and collate the emerging evidence around DLUS education, training, governance, and implementation, to aid the future adoption of DLUS into therapist’s practice. Readers can find all the included studies listed in Supplementary File 1.

## Methods

This scoping review was conducted following the guidelines of the Preferred Reporting Items for Systematic Reviews and Meta-analysis protocol extension for scoping reviews (PRISMA-ScR) [31]. No patient or personal data was included, and the scoping review did not require ethical approval.

The Participants, Concept and Context (PCC) method [32] was employed to formulate the following research question:

“In what ways have physiotherapists, physical therapists or respiratory therapists adopted lung and/or diaphragm ultrasound to inform their research, clinical and educational practice?”

**P (Participants)**—Physiotherapists, physical therapists or respiratory therapists as study participants, adult patients, paediatric patients, or healthy volunteers.

**C (Concept)**—The use of lung ultrasound (LUS) and/or diaphragm ultrasound (DUS) involving a physiotherapist/physical therapist/respiratory therapist.

**C (Context)**—Publications from educational, clinical or research settings where a physiotherapist/physical therapist/respiratory therapist was the first author.

### Information sources

Search strategies drafted by co-author and clinical librarian (MR) were refined through two pilot searches in conjunction with team discussions (Supplementary File 2). Bibliographic databases searched electronically were AMED, EmCare, CINAHL, Embase, Medline, PubMed and Pedro. Search results were exported to Excel and duplicates removed. Search dates were from 1997 to December 2023: as the previous scoping review in 2018 found no relevant papers prior to 1997. The search had no language restrictions.

Reference lists of included studies were searched and the ‘related articles’ function in PubMed was utilised. Clinical trial registries were searched, including ClinicalTrials.gov, the International Clinical Trials Registry Platform and Current Controlled Trials meta-Register of Controlled Trials. Citation lists from key papers in this area of study were examined for relevant publications.

Grey literature: Google Scholar, The Association of Respiratory Physiotherapists in Respiratory Care (ACPRC) journal (from the Chartered Society of Physiotherapists, United Kingdom), TRIP database, OpenGrey, Bielefeld Academic Search Engine and university repositories were searched.

Author correspondence: Twenty-seven key authors, known to publish in this area of study, were contacted for any additional published work.

### Eligibility criteria

All empirical publications in any language, including conference abstracts, were included (unless a corresponding full paper publication was available). Reviews, editorials, letters, commentaries, informal interviews, or opinion publications were excluded. Publications studying humans in utero or animal studies were excluded.

### Selection of sources of evidence

**Fig. 1:**
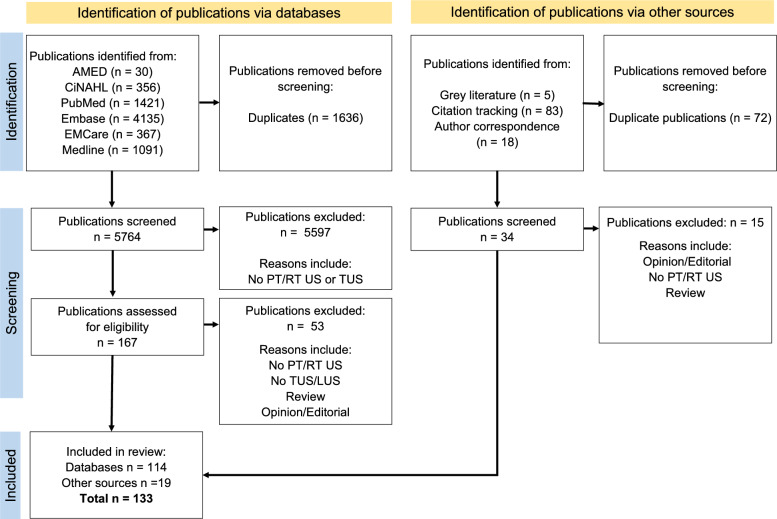
Study selection PRISMA-ScR flow diagram. (PT: physical therapists/physiotherapists, RT: respiratory therapists, US: ultrasound, DLUS: diaphragm or lung ultrasound)

Two independent reviewers (SH and CC) screened titles and abstracts for inclusion. Those that did not meet the Participant, Concept and Context (PCC) criteria, or the exclusion criteria were removed. Full text versions of remaining studies were obtained and screened by the same two reviewers to determine eligibility. Disagreements were resolved by a third author (CT). Reasons for exclusion were noted and the process of study selection is documented Fig. 1: Study selection PRISMA-ScR flow diagram, as per PRISMA-ScR recommendations [33].

### Data charting

To increase consistency between reviewers a data extraction and charting form was developed and piloted by four authors (SH, CT, CC, and AM) on three full text publications. The final version of the form can be found in Supplementary File 3. Disagreements on study selection and data extraction were resolved through discussions between authors. Studies published in a language other than English were reviewed by either a fluent French speaker (CC) or a fluent Spanish/Portuguese speaker (AM). One study published in Korean was translated via an on-line translation website [34] to chart the relevant information.

Extracted information included: title, author(s), full citation, year of publication, country of origin, professional group involved (population), lung or diaphragm ultrasound (concept), evaluation method, educational, clinical or research setting (context), subject/disease/patient group, sample size, study design and professional group performing the DLUS.

As is common with scoping reviews, no assessment of the quality of included publications has been performed. However, comment will be made on the overall quality of the evidence-base for DLUS studies (both DUS and LUS) when compared to the hierarchy-of-evidence. The full list of included studies and their extracted characteristics can be found in Supplementary File 1.

## Results

A total of 7506 potential studies were identified in the database searches. Following the removal of duplicates, the remaining abstracts were evaluated for relevance to the research question and screened against the inclusion and exclusion criteria. A total of 7286 studies were excluded (Fig. 1) with 220 studies being obtained in full publication format and assessed for their eligibility. Following all screening and assessment, 133 studies met all inclusion criteria for this scoping review. Of the 133 studies, 104 were available as a full publication and 29 were available as a conference abstract without a corresponding full publication. Included studies were published over a 26-year period between the years of 1997 and 2023.

### Geographical location

**Fig. 2:**
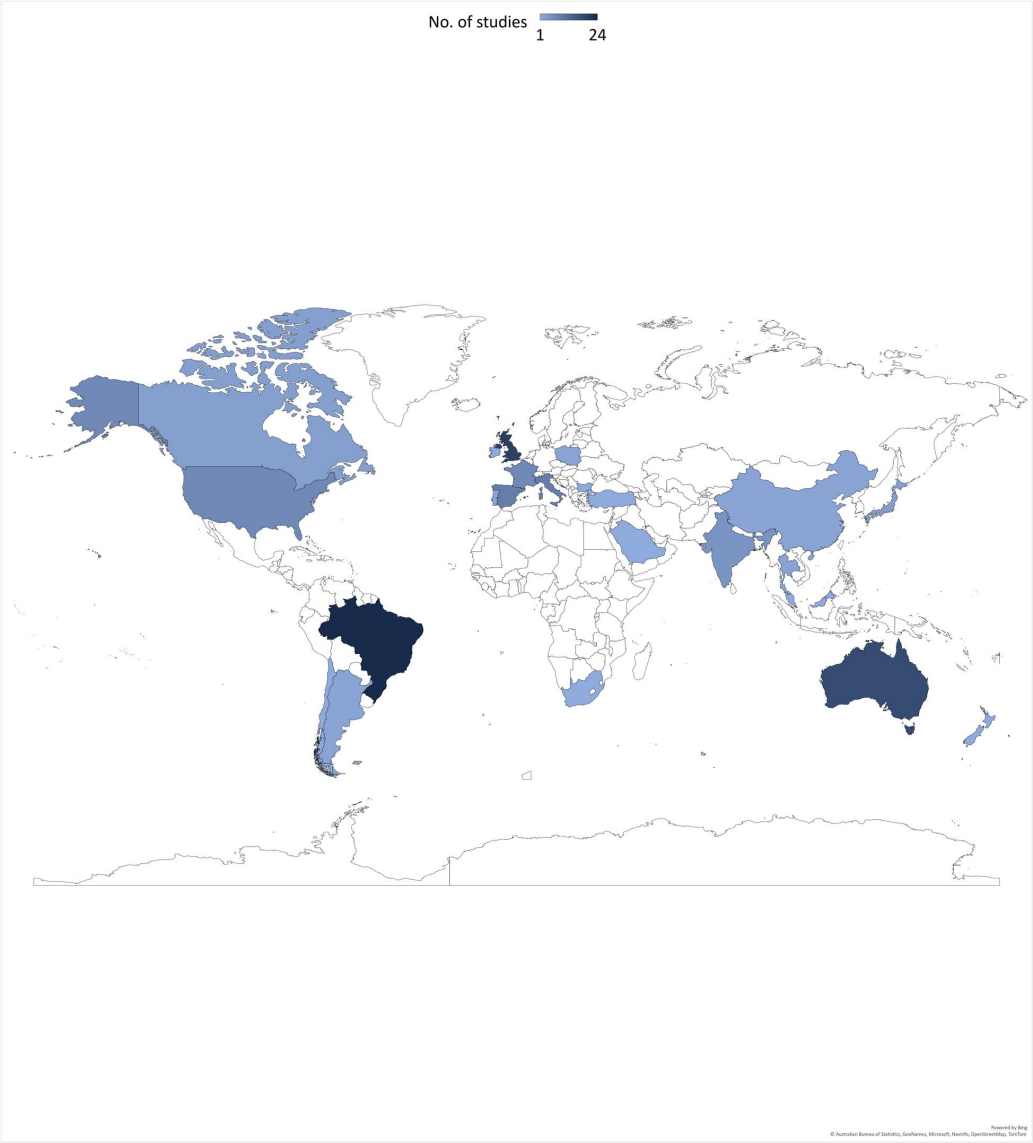
Geographical location of included studies

The 133 included studies were completed in 26 different countries (Fig. 2). Three countries accounted for almost half the included studies (46%): Brazil (n = 24), the United Kingdom (U.K.) (n = 20) and Australia (n = 18).

### Participants

A total of 89 studies were either completed by, or involved physiotherapists as the study participants, whilst physical therapists and respiratory therapists contributed to a total of 26 and 18 studies respectively.

The 133 included studies included 27 different participant populations with the 10 most frequently studied populations being listed in Table 1. With 43 studies in total, patients suffering from critical illness were the most studied population, for both DUS and LUS, followed by healthy volunteers (n = 16) and physiotherapists as study participants (i.e. educational or surveys studies) (n = 12). Together, these three participant populations account for almost half of the included studies.

**Table 1:** The 10 most frequently studied participant populations

Participant population	Numbers
Patients with critical illness	43
Healthy volunteers	16
Physiotherapists (participants in educational or survey studies)	12
Patients with Chronic Obstructive Pulmonary Disease (COPD)	10
Respiratory therapists (participants in educational or survey studies)	9
Patients with respiratory disease (excluding COPD)	9
Patients after surgery	5
Patients with COVID-19	4
Athletes	4
Patients following a cerebral vascular accident (CVA)	3

This scoping review highlighted that an additional 17 populations have now been investigated by therapists with DLUS. All 27 participant populations can be found in Supplementary File 1.

### Concept

A total of 71 studies reported on the use of DUS, 58 reported on the use of LUS and 4 reported on both DUS and LUS. The earliest DUS study was published in 1997 while the first LUS study was published in 2014 as a conference abstract.

**Fig. 3:**
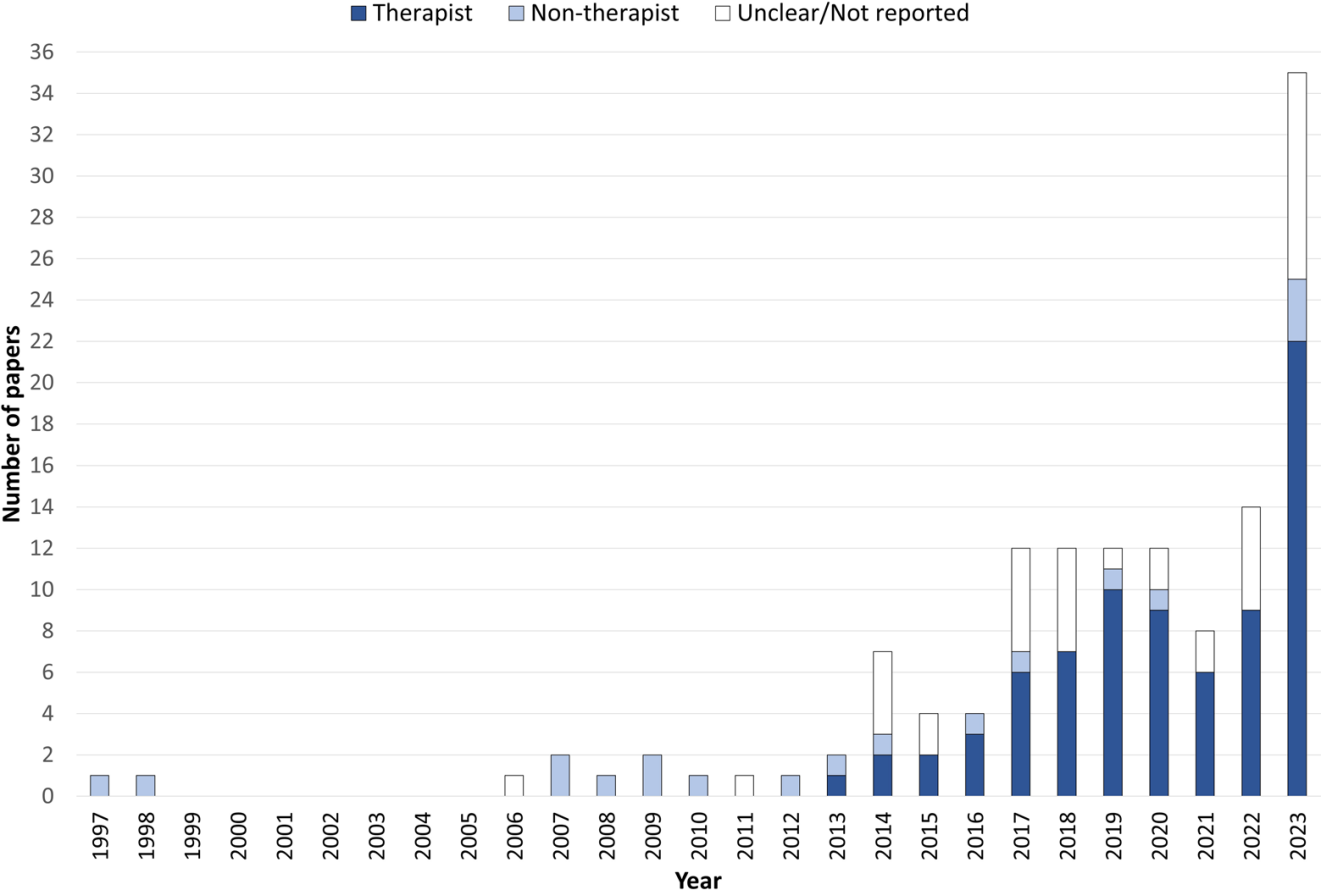
Profession performing the DLUS by year *(n* = *133)*

In over half of the included studies (n = 77), a therapist was reported as the profession specifically performing either the DUS or LUS. The background of the profession performing the DLUS was reported as a non-therapist (i.e. sonographer) in 18 of the studies, while the profession was either not reported or was unclear in the remaining 38 studies (Fig. 3).

The most reported method of diaphragm evaluation was excursion found in 45 of the DUS studies. As for the method of LUS evaluation, the 6-zone and 12-zone protocols were reported equally in 14 studies each.

### Context

The use of DLUS in a research context contributed the largest proportion of included studies (n = 69), followed by clinical (n = 46) and finally educational (n = 18).

The most frequent methodology used was observational (n = 50) with the greatest proportion being cross-sectional (n = 23) and prospective cohort (n = 17) studies. A total of 41 studies used experimental methodology with 13 randomized control trial (RCT) study designs. Case studies and case series contributed to nearly a quarter of all studies (n = 31). Qualitative studies included in the results comprised of 6 surveys and 1 interview, primarily investigating therapist’s experiences or perceptions of training, adopting, implementing, or utilising LUS in their practice.

## Discussion

Using an enhanced search strategy, this scoping review updated the evidence around the adoption of DLUS by therapists. In the last 7 years there has been a five-fold increase in the number of studies (n = 26 to n = 133).

### Geographical location

The publication of DLUS studies now has international contributions from 26 countries (Fig. 2), with the top three publishing countries (Brazil, the U.K. and Australia) being in three separate continents. The number of countries publishing therapist led DLUS research (n = 26) has almost tripled in number since the previous scoping review which included publications from 9 different countries [20]. However, all studies were published by authors from middle- or high-income countries meaning low-income countries [35] that could also benefit from the adoption of DLUS, are not represented and should be included in future work.

### Participants

As with the previous scoping review [20], study participants experiencing critical illness or those with COPD represented a high proportion of the patient populations being investigated (Table 1). In contrast, the increased recruitment of healthy volunteers and therapists themselves as participants into these studies appears to suggest the development of baseline metrics regarding DLUS, and the exploration of therapist specific factors in the adoption of DLUS into practice respectively. The participant category “Patients with critical illness” includes a wide range of pathologies or conditions and would benefit from being divided into further subcategories in the future such as lung consolidation, pleural effusion or diaphragm dysfunction.

With the number of participant populations increasing from 10 to 27, the utilisation of DLUS to investigate numerous varied presentations, conditions and diseases is clearly growing.

However, less than 10% of the studies (n = 12) investigated therapists’ use of DLUS in a paediatric population. This suggests a need for additional work into this population especially considering the added safety benefits of ultrasound when compared to the ionising radiation exposure of a chest radiograph or thoracic computed tomography.

### Concept

An additional 107 studies have been published between 2016 and 2023, with 35 having been published in 2023 alone (Fig. 3). This figure is more than double that of 2022 (n = 14) showing a dramatic increase in the number of research publications from therapists. Despite the number of published studies for both LUS and DUS increasing, the largest growth was seen with LUS where the number of studies went from n = 3 in 2016, to n = 58 by the end of 2023 thus showing the continued interest in this newer application of ultrasound imaging.

Even with this rapid increase of 107 studies in the last 7 years, the proportion of studies reporting that a therapist performed the ultrasound scanning themselves, has remained consistent at over 50% of the included studies (Fig. 3). This proportion could potentially be higher, but the lack of detail in reporting makes any further clarification difficult. It is recommended that both the professional background and the scanning experience of those performing the ultrasound should be included in future studies. The main point here is that as the research around DUS and LUS grows, therapists are actively participating in the performing of the ultrasound scans themselves, and not relying on other professions to perform the scans for them. This would allow therapists to perform ultrasound scans in line with the values of wider point-of-care ultrasound (POCUS) utility through therapist specific ultrasound indications, acquisition, interpretation and integration into the clinical reasoning process for enhanced patient management [36].

### Context

The use of DLUS in a research capacity account for over half the included studies (52%), whether being investigated for its reliability and validity as a therapist-performed imaging tool, or as an outcome measure to investigate other treatments/interventions. Almost a third of the included studies (34%) reported on the use of DLUS as an imaging tool within clinical practice, thus highlighting the increased uptake of DLUS to enhance pathology or dysfunction identification, aid clinical reasoning towards optimal patient management, and evaluating the effectiveness of therapist’s treatments and interventions.

With the earliest search results for therapist studies in DUS and LUS having been published in 1997 and 2014 (respectively), the therapist evidence-base for DUS has been established 14-years longer than LUS. This difference is more apparent when comparing the respective included studies against the hierarchy-of-evidence [37]. Overall, DUS has a consistently higher level of evidence with most study methodologies being experimental, including 12 of the 13 RCT found (Supplementary File 1). In contrast, a large proportion of the evidence for LUS is situated lower down the hierarchy (case report/series) with fewer experimental or observational studies published (Supplementary File 1). Progress has been made in the quality of evidence for both DUS and LUS since the previous scoping review, however, focused efforts to enhance the methodological rigor of future studies, particularly for LUS, is recommended.

The high degree of heterogeneity between studies across the extracted data categories (study design, participants, disease conditions) suggests that this imaging modality remains in its infancy as all three therapist professions explore the potential adoption of DLUS within their respective research, clinical and educational practice. It is hoped that this scoping review may facilitate some degree of collaboration and coordination between these professional groups. Collaboration between the three therapy professions could benefit all, considering many aspects of research, clinical and educational practice for DLUS have some overlap.

Importantly, the higher number (n = 18) of included studies investigating education and training is encouraging (Supplementary File 1), especially alongside the emerging research outputs incorporating the governance and implementation of LUS (Supplementary File 1). In contrast, there was no published evidence around the education, adoption or implementation of DUS into therapist’s practice. The adoption of both DUS and LUS into a therapist’s practice should involve robust training processes and governance structures to guarantee high levels of therapist skill and quality assurance [34]. The framework from Smith et al. [38] in conjunction with two documents from the Chartered Society of Physiotherapists (CSP) in the U.K. on the context [39] and guidance [40] on POCUS in physiotherapy practice, may give readers the confidence to begin navigating the governance of DLUS adoption for their own practice within the healthcare systems at their geographical location.

### Potential research areas

Many aspects of the use of DLUS by therapists still warrant further investigation, so listed below are some potential research gaps in the DLUS literature. Please note, this list is not exhaustive.

- Financial cost versus benefit of therapist performed DLUS.
- Engagement in the research and use of DLUS in low-income countries.
- Additional work in the neonatal and paediatric populations.
- Enhance the methodological rigor of future studies, particularly for LUS.
- Additional DLUS work in respiratory interventions (i.e. inspiratory muscle training)
- Expand participant populations for DUS (i.e. neuromuscular diseases)
- Expand participant populations for LUS.

Robust methods of training in and adoption of DLUS needs to continue, especially as the uptake of DLUS appears to be growing internationally [1–5]. There is still a need to show how both DUS and LUS imaging techniques affect patient outcomes, as well as the financial cost versus benefit of therapist performed DLUS.

## Limitations

Studies published in databases not included in the search strategy may have been missed. Not all studies will have identified or stated that a physiotherapist, physical therapist, or respiratory therapist was involved as either first author, corresponding author or as a participant. Nonetheless, multiple strategies were employed to minimise this risk. The methodological quality of some included publications retrieved from the grey literature may not be as robust due to a lack of an accepted peer-review process.

## Conclusion

The number of DLUS studies involving therapists continues to show international growth with a very large increase in published studies in 2023 alone. Studies are investigating an ever-increasing range of participant populations with differing methodologies showing the current and potential diverse uses of DLUS in the hands of therapists. Published studies now include research on LUS adoption, implementation, and utility amongst all three of the therapy professions, however, DUS remains lacking in this area. The potential of DLUS and its impact on patients relating to diagnosis, monitoring interventions, patient specific outcomes, and its long-term outcomes on society, still need to be explored. Regardless, DLUS remains a novel and innovative imaging technique in the hands of physiotherapists, physical therapists, and respiratory therapists as its utility continues to grow in various research, clinical and educational settings.

## Supplementary Information

Supplementary material 1. Excel spreadsheet of all 133 included studies.

Supplementary material 2. Database search strategy.

Supplementary material 3. Data charting form.

## Data Availability

All data generated or analysed during this study are included in this published article and its supplementary information files.

## Abbreviations

DLUS: Diaphragm and lung ultrasound
TUS: Thoracic ultrasound
AMED: Allied and complementary medicine database
CINAHL: Cumulated index to nursing and allied health literature
PCC: Participant, concept, context
PRISMA-ScR: Preferred reporting Items for systematic reviews & meta-analysis protocol extension for scoping reviews
LUS: Lung ultrasound
DUS: Diaphragm ultrasound
COVID-19: Coronavirus disease caused by the SARS-CoV-2 virus
ACPRC: Association of chartered physiotherapists in respiratory care
TRIP database: Turning research into practice database
U.K: United Kingdom
COPD: Chronic obstructive pulmonary disease
CVA: Cerebral vascular accident
RCT: Randomised control trial
CSP: Chartered Society of Physiotherapists
POCUS: Point of care ultrasound
