# Supplementary material for: Exploring the adoption of diaphragm and lung ultrasound (DLUS) by physiotherapists, physical therapists, and respiratory therapists: an updated scoping review
Source: Ultrasound J. 2025 Jan 20;17:9. doi: 10.1186/s13089-025-00412-w (PMC11747032; doi:10.1186/s13089-025-00412-w)
Supplement: Supplementary file 2 — Supplementary material 2. Database search strategy. [file 13089_2025_412_MOESM2_ESM.docx]

**Supplementary material 2 - Database search strategy**

P (Participants): physiotherapist – physio*

PHYSIOTHERAPY – MeSH Heading

PHYSIOTHERAPIST – MeSH Heading

physical therapist – physical AND therap*

respiratory therapist – respiratory AND therap*

kinesitherap*

kinesiotherap*

KINESIOTHERAPY – MeSH Heading

RESPIRATORY THERAPIST or
 RESPIRATORY THERAPY or
 RESPIRATORY THERAPY DEPARTMENT, HOSPITAL – MeSH Heading

C (Concept): Lung – lung OR lungs

LUNG – MeSH Heading

Thoracic – thora*

Chest – chest

THORAX – MeSH Heading

Pulmonary – pulmon*

Respiratory – respirat*

Diaphragm – diaphragm* or diafragm

DIAGPHRAGM – MeSH Heading

Ultrasound – ultraso*

ULTRASOUND – MeSH Heading

Sonography – sonogra*

echogra*

ECHOGRAPHY – MeSH Heading

C (Context): Any publication type in any educational, clinical or research setting
