# Supplementary material for: Exploring the adoption of diaphragm and lung ultrasound (DLUS) by physiotherapists, physical therapists, and respiratory therapists: an updated scoping review
Source: Ultrasound J. 2025 Jan 20;17:9. doi: 10.1186/s13089-025-00412-w (PMC11747032; doi:10.1186/s13089-025-00412-w)
Supplement: Supplementary file 3 — Supplementary material 3. Data charting form. [file 13089_2025_412_MOESM3_ESM.docx]

**Supplementary material 3 - Data charting form**

**Physiotherapy thoracic/lung ultrasound scoping review data charting form**

| **Scoping Review Details** | |
| --- | --- |
| Scoping Review Title: | Use of thoracic/lung ultrasound by physiotherapists: a scoping review |
| Scoping Review Objectives: | To explore and map out the current evidence base around the adoption of TUS by physiotherapists, physical therapists, and respiratory therapists and how this imaging modality informs their research, clinical and educational practice. |
| Scoping Review question: | In what ways have physiotherapists, physical therapists or respiratory therapists adopted thoracic (lung and/or diaphragm) to inform their research, clinical and educational practice? |
| **Inclusion/Exclusion Criteria** | |
| Population | Physiotherapist, Physical Therapist (PT) or Respiratory Therapist (RT) |
| Concept | Pleural & lung or diaphragm ultrasound |
| Context | Any including (but not limited to) education, clinical practice or in a research capacity |
| **Evidence source details/characteristics** | |
| Title & Citation |  |
| Author(s) |  |
| Year of publication |  |
| Country of origin |  |
| Population: (Physio/Physical Therapist/Respiratory Therapist) |  |
| Concept  (Lung/diaphragm/both) |  |
| Context  (Educational, clinical, research) |  |
| Subject/disease/patient group |  |
| Sample size |  |
| Study design |  |
| Profession performing the US |  |
